# The role of miR‐128 and MDFI in cardiac hypertrophy and heart failure: Mechanistic

**DOI:** 10.1111/jcmm.18546

**Published:** 2024-07-24

**Authors:** Sun Yanjun, Gu Yunfen, Yao Haoyi, Wang Zhe, Qiu Jiapei

**Affiliations:** ^1^ Department of Cardiovascular Surgery Ruijin Hospital, Shanghai Jiao Tong University School of Medicine Shanghai China; ^2^ Department of Intensive care unit, Ruijin Hospital Shanghai Jiaotong University School of Medicine Shanghai China

**Keywords:** apoptosis, cardiac hypertrophy, cardiomyocytes, heart injury, MDFI, miR‐128‐3p, MyoD, proliferation, Wnt/β‐catenin pathway

## Abstract

Heart failure (HF) prognosis depends on various regulatory factors; microRNA‐128 (miR‐128) is identified as a regulator of cardiac fibrosis, contributing to HF. MyoD family inhibitor (MDFI), which is reported to be related with Wnt/β‐catenin pathway, is supposed to be regulated by miR‐128. This study investigates the interaction between miR‐128 and MDFI in cardiomyocyte development and elucidates its role in heart injury. Gene expression profiling assessed miR‐128's effect on MDFI expression in HF using qPCR and Western blot analysis. Luciferase assays studied the direct interaction between miR‐128 and MDFI. MTT, transwell, and immunohistochemistry evaluated the effects of miR‐128 and MDFI on myocardial cells in mice HF. Genescan and luciferase assays validated the interaction between miR‐128 and MDFI sequences. miR‐128 mimics significantly reduced MDFI expression at mRNA and protein levels with decrease rate of 55%. Overexpression of miR‐128 promoted apoptosis with the increase rate 65% and attenuated cardiomyocyte proliferation, while MDFI upregulation significantly enhanced proliferation. Elevated miR‐128 levels upregulated Wnt1 and β‐catenin expression, whereas increased MDFI levels inhibited these expressions. Histological analysis with haematoxylin and eosin staining revealed that miR‐128 absorption reduced MDFI expression, hindering cell proliferation and cardiac repair, with echocardiography showing corresponding improvements in cardiac function. Our findings suggest miR‐128 interacts with MDFI, playing a crucial role in HF management by modulating the Wnt1/β‐catenin pathway. Suppression of miR‐128 could promote cardiomyocyte proliferation, highlighting the potential value of the miR‐128/MDFI interplay in HF treatment.

## 1 INTRODUCTION

Heart failure (HF) is a complex condition affecting millions of people worldwide; its prognosis depends on many factors, such as the cause of HF, how well the heart is functioning, and how well the patient is managing their condition.^1^, ^2^ Cardiac hypertrophy represents one of the most recurrent adaptive mechanisms deployed by chronic heart inflammation.^3^ Typically, the heart's enlargement can manifest as an expansion of the left ventricular cavity, a condition termed ‘asymmetric hypertrophy’, or it may present as a thickening of the ventricular walls, referred to as ‘concentric hypertrophy’. These structural alterations are compensatory response to increased pressure or volume load on the heart. It is increasingly acknowledged that such hypertrophy changes, when they become maladaptive, are a key factor in the advancement of cardiac impairment, which can precipitate the onset of HF.^4^

Emerging research has suggested the participation of miR‐128, a small non‐coding microRNA, in myocardial infarction pathogenesis. It is particularly remarkable that levels of miR‐128‐3p were significantly reduced in the serum of patients with atherosclerosis (AS). Furthermore, miR‐128‐3p has been shown to attenuate inflammatory activity in VSMCs and curb their dysregulated proliferation and migration.^5^ Another study demonstrated that miR‐128 is upregulated during the progression of HF, leading to increased deposition of extracellular matrix proteins in the heart and increased cardiac fibrosis. Furthermore, studies have demonstrated that Tongxinluo can downregulate miR‐128‐3p expression, thereby shielding human cardiomyocytes against ischemic damage by activating the p70s6k1/p‐p70s6k1 signalling cascade.^6^ It has been proposed that miR‐128 is involved in the regulation of the Wnt/β‐catenin pathway which is important for regulating cell proliferation, differentiation, and gene expression and is essential for cardiac development and maintenance.^7^, ^8^ Wnt/β‐catenin pathway regulate the expression of the myogenic factor MyoD, also which plays an important role in the regulation of cardiac muscle cell differentiation and growth, suggesting that miR‐128 may play a role in the development of HF by regulating Wnt/β‐catenin pathway pathways.^9^, ^10^ However, further research is needed to identify the exact mechanisms by which miR‐128 contributes to the development of HF.

MyoD is a transcription factor that plays an important role in the development and maintenance of skeletal muscle. It is also known to be involved in the repair and regeneration of damaged skeletal muscle tissue, which may be important in the HF setting. In mice with HF, MyoD has been shown to be upregulated and believed to be involved in the process of myocardial repair and regeneration, which may be beneficial in helping to improve cardiac function.^11^ There are currently no known MyoD family inhibitors (MDFIs) that have been identified as having an effect on cardiomyocytes. However, there are a variety of compounds that have been identified as modulators of MyoD family members in other tissue types that may have an effect on cardiomyocytes.

Research has revealed that the inhibitor of the MyoD factor, termed MDFI or I‐mf, negatively influences the transcriptional governance of MyoD gene members in the course of myogenic differentiation in NIH‐3T3 cells. MDFI is upregulated in Sol8 cells but downregulated in HL1 cardiomyocytes by miR‐27 overexpression.^12^ Interestingly, MDFI overexpression aided in the transition of type IIb muscle fibres into type IIa and type I muscle fibres and myogenic development of C2C12 cells. Nevertheless, it is uncertain how its interaction with downstream miRNAs affects their expression.^13^ Additionally, it was recently proved that the overexpression of miR‐27b considerably enhanced PSCs myogenesis by sponging the expression of MDFI. The above literature suggests that MDFI may have huge potential for finding the updated therapy for patients with the muscle injury.^14^ Therefore, it becomes essential to investigate the relationship between MDFI and miRNAs involved in heart injury.

In the current work, utilizing isolated cardiomyocytes cell lines and animal models of isoproterenol (ISO) infusion, our research elucidated the detrimental role of the miR‐128/MDFI interplay in advancing cardiac hypertrophy and HF, through the upregulation of the Wnt1/β‐catenin signalling axis.

## 2 METHODS

### 2.1 Cell culture and transfection

Cardiomyocytes were isolated from neonatal mice using a specific kit (Cellutron Life Technology, USA). Cardiac tissues harvested from 2 to 3 days old C57BL/6 mice were sterilely procured, weighed, sectioned and enzymatically broken down under agitation at 37°C for a duration of 12 min. Subsequently, the resultant cell homogenates were centrifuged at a speed of 1200 rpm for further processing. The collected cells were incubated for 24 h at 37°C with 5% CO₂. As delineated in earlier reports, cardiomyocytes were subjected to shRNA‐MDFI to diminish MDFI gene expression, concurrently, to replicate the full MDF1 gene, the pCDH‐CMV‐MCS‐EF1A‐GFP‐T2A‐Puro vector (System Biosciences) was utilized, generating an overexpression vector termed MDF1‐OE. Cells cultured to 60%–70% confluence received the plasmids for transfection, employing Lipofectamine® 2000 (Invitrogen; Thermo Fisher Scientific) as per the manufacturer's instructions. Once the cells attained 60%–70% confluence, a transfection mix containing 4 μg each of PCDH and PLVX‐shRNA1 vectors, along with 500 mL of Opti‐MEM and 3.5 mL of Lipofectamine® RNAiMAX (Invitrogen; Thermo Fisher Scientific) was prepared with the shRNA and pCDH‐MDFI OE vectors and then deposited onto a 3.5 cm culture dish. Forty‐eight hours post‐application, the efficacy of gene delivery was assessed via flow cytometry, RT‐qPCR, and immunoblotting techniques.

### 2.2 CCK‐8 essay

A Cell Counting Kit 8 (CCK‐8) test was performed in accordance with the package recommendations to measure the viability of HL1 cells (Beyotime Institute of Biotechnology). HL1 cells were seeded into 96‐well plates at a density of 1 × 10^^5^ cells per well and incubated for 24 h. Subsequently, at the 72‐h mark, each well received 10 μL of the CCK‐8 reagent. The absorbance was measured using a spectrophotometer at a 450 nm wavelength after incubating the wells for 4 h. Cell viability was calculated using the following formula: percentage of cell viability = [(average absorbance of the treated HL1 cells)/(average absorbance of the untreated HL1 cells)] × 100%.

### 2.3 Crystal violet staining

Briefly stated, cells were initially plated at 1 × 10^^6^ in MEM containing 10% FBS, 10 U/L penicillin, and 10 g/L streptomycin (upper chamber). Following a 48‐h incubation at 37°C, cells were fixed in 4% paraformaldehyde for 4 h at 25°C and subsequently stained with 0.5% crystal violet for 15 min. The density of cells was quantified in five selected fields using a microscope (CX43, Olympus Corporation).

### 2.4 Flow cytometry

Following the assembly specifications, flow cytometry was used to assess cell apoptosis using an Annexin V‐FITC/PI apoptosis kit from BD Biosciences. To generate a cell suspension (1 × 10^6^ cells/mL), 100 μL of binding buffer was introduced after cell harvesting. Following a 15‐min room temperature incubation with FITC‐Annexin V and PI, to prepare flow cytometry, cells were then suspended in 400 μL binding buffer (FACSCalibur, Bio‐Rad). Using the program FlowJo 7.6.1, the number of cells in early and late apoptosis was measured (FlowJo LLC).

### 2.5 Immunoblotting

HL1 cardiomyocytes and mouse cardiac tissues were lysed using RIPA buffer (Beyotime Biotechnology), which was enriched with protease and phosphatase inhibitors, to extract proteins for western blot analysis (Beyotime Biotechnology). The BCA protein detection kit was used to measure protein concentrations (Beyotime Biotechnology). 30 μg of protein, in equal portions, was isolated using 10% SDS‐PAGE and then applied to PVDF membranes. The membranes were treated at 4°C overnight with primary antibodies directed against GAPDH (ab9485; Abcam), Wnt1 (ab85060; Abcam), catenin (ab196204; Abcam), and rabbit polyclonal antibody to MDFI (A99387) after blocking with 5% skim milk powder for 1 h at 25°C. The membranes were then treated for 1 h at room temperature with the matching secondary antibodies [Goat Anti‐Rabbit IgG; ab6721, 1:1500; Abcam]. Following this, proteins were identified through enhanced chemiluminescence as instructed by the supplier (Beyotime, China).

### 2.6 Animal experiments

Male C57BL/6 mice were 8–10 weeks old and 20–25 g were obtained from GemPharmatech Nanjing, China. Adhering to the Ruijin Hospital Ethics Committee's guidelines from Shanghai Jiao Tong University School of Medicine (ethics committee certification number: RJH202202033), the mice were raised in a controlled pathogen‐free setting, with regulated temperature and humidity levels with a constant temperature of 21°C and 12:12‐h dark–light cycle. Animals were randomly divided into experimental group and control group.

All C57BL/6 mice were divided into six groups (*n* = 3), PBS‐control, PBS‐anti‐mir‐128, PBS‐anti‐mir‐128 + MDFI‐OE, ISO‐control, ISO‐anti‐mir‐128, ISO‐anti‐mir‐128 + MDFI‐OE. For ISO infusion, mice were implanted with mini‐osmotic pumps (Alzet models 1004 and 1002, DURECT Corp. CA) and given continuous infusions of isoproterenol (30 mg/kg/day, Sigma‐Aldrich) for a total of 14 days to cause chronic HF. And the control groups were given PBS instead. Genes are transferred through adenovirus by injected into the myocardium. The injection dose of adenovirus was 2 × 10^11^ pfu/mL. After 5 days of injection, myocardial function were monitored and recorded. Then, after anaesthesia, the myocardial tissue from the injured area were token and stored properly.

### 2.7 Echocardiography

Mice were supplied with oxygen at a flow rate of 1 L/min supplemented with 1.0% isoflurane for inhalation. Cardiac function was evaluated using an 18–38 MHz MS400 transducer connected to a Visualsonic Vero 2100 echocardiography system. Consistent imaging was achieved by standardizing to an M‐mode short‐axis view at the LV mid‐papillary level, enabling accurate measurement of cardiac parameters such as EF, FS and LVID. For each heart, two‐dimensional images encapsulating at least five successive cardiac cycles were captured to guarantee reliable measurement and analysis.

### 2.8 Haematoxylin and eosin staining

About 1–3 cm^3^ heart tissues were isolated and fixed in 4% paraformaldehyde for 3 days. Tissue sections were then cut at 5 μm thickness and processed for gradient paraffin embedding. Slides were stained with haematoxylin and eosin following standard protocols, and imaged with an Olympus BH 2 photomicroscope.

### 2.9 Dual‐luciferase reporter assay

Wild‐type and mutant sequences, predicted to be miR‐128's binding regions in MDFI mRNA's 3'UTR, were confirmed and engineered into a pGL3‐reporter vector. The rAAV9 system was then applied to administer MDFI‐luc reporter constructs with either control vectors or miR‐128 mimics into mice. At 4 weeks after transfection, harvested cardiomyocytes were processed using a Firefly Luciferase Assay Kit according to Beyotime's instructions. We measured the luciferase signal from these preparations using a Glomax20/20 luminometer, with renilla luciferase serving as a baseline for luciferase signal comparison.

### 2.10 Quantitative real‐time PCR

After corresponding treatment of myocardial cells, we used TRIzol reagent to isolate the total messenger RNA. Subsequently, using an EasyScript First‐Strand cDNA synthesis Supermix (TransGen Biotech, China), 1 μg of total RNA was reverse‐transcribed into cDNA in accordance with the manufacturer's instructions. A standard LightCycler 480 SYBR Green I Master procedure was used to produce quantitative real‐time PCR (qRT‐PCR), which was then run in triplicate and evaluated using a LightCycler 96 System (Roche, Switzerland). The Ct value was used to standardize the relative expression levels of target genes by using GAPDH as a reference gene. The following primers were used for qRT‐PCR: miR‐128 forward 5′‐GGTCACAGTGAACCGGTC‐3′ and reverse 5′‐GTGCAGGGTCCGAGGT‐3′; MDFI forward 5′‐GAGCGGTCAGTGCCCTTCT‐3′ and reverse 5′‐CAGGGTGAGTGGATCTTGCTAC‐3′.

### 2.11 Statistical analysis

Utilizing GraphPad Prism (GraphPad Software Inc., La Jolla, CA, USA), all data were represented as means with structural equation modelling (SEM), for the number of samples was relatively small, with *n* = 3. The significance of the variance between the groups was assessed using the unpaired, two‐tailed Student *t* test, or the one‐way analysis of variance (ANOVA). Statistics were deemed significant at *p* < 0.05.

## 3 RESULTS

### 3.1 miR‐128 expression increase cell apoptosis and suppresses cell proliferation and viability

**FIGURE 1:**
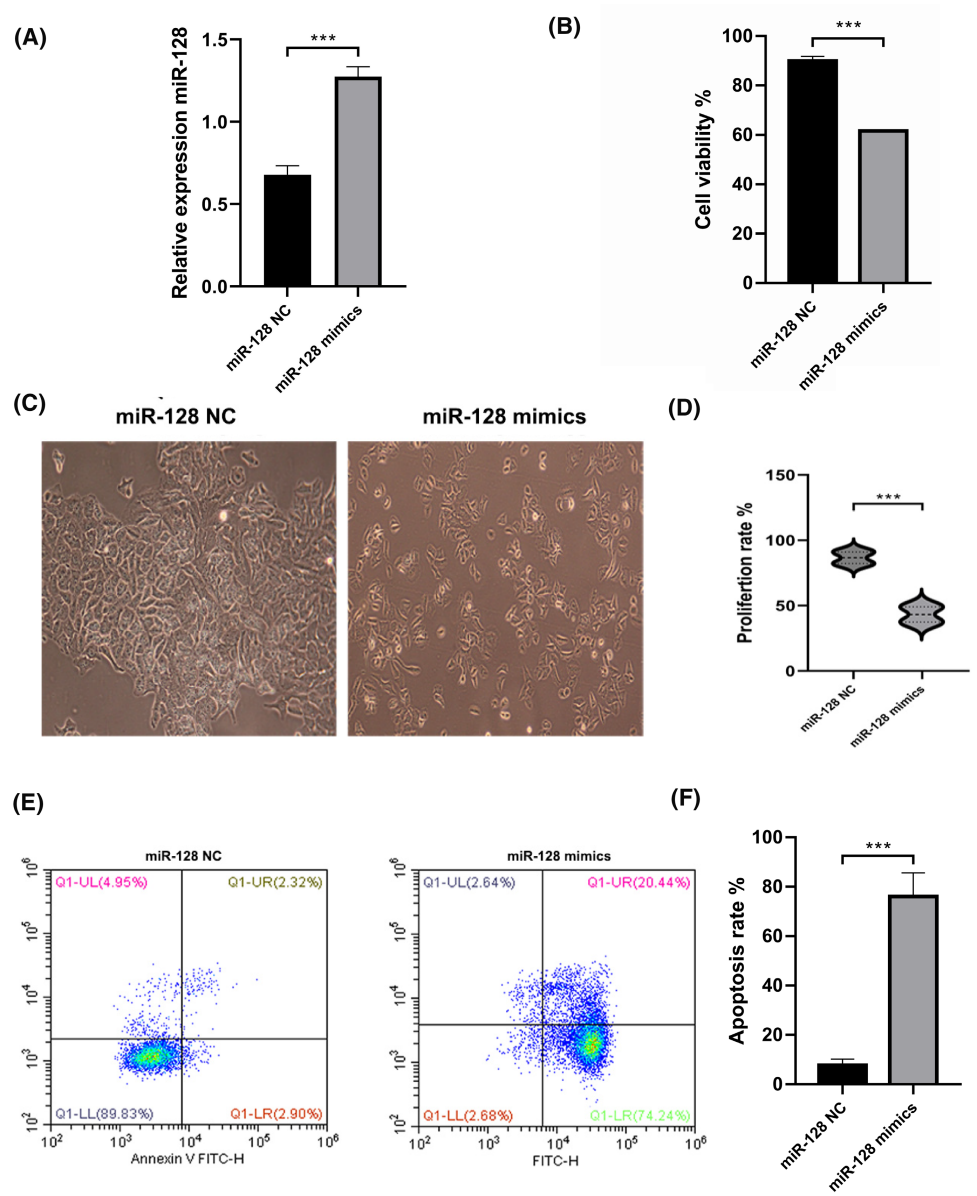
miR‐128 expression increases cell apoptosis and suppresses cell proliferation and viability. (A) qPCR showing upgraded expression of miR‐128‐3p in miR‐128‐mimics cells; (B) overexpression of miR‐128‐3p significantly reduces the cell viability; C and D) HL1 cell viability (magnification, ×100); (E, F) overexpression of miR‐128‐3p enhances cardiomyocytes apoptosis; data are presented as means with ±SEM of triplicate samples. *n* = 3. ***p < 0.001 compared with control group.

To investigate the role of miR‐128 in the development of HL1 cells, flow cytometry, MTT and transwell essays were subsequently carried out in HL1 cells transfected with miR‐128 negative control and miR‐128‐mimics. First, our qPCR and western blot results confirmed the successful transformation of HL1‐miR‐128‐mimics with an upgraded expression of miR‐128 at mRNA levels by QPCR (Figure 1A). Second, when comparing with the control, the overexpression of miR‐128 significantly reduced the cell viability rate among the HL1‐treated cells, while the flow cytometric results exhibited an enhanced level of apoptotic cells in response to miR‐128 mimics treatment (Figure 1B–F). These results demonstrated that the upregulation of miR‐128 promotes apoptosis and inhibits the proliferation of cardiac myocyte cell lines, confirming its pivotal role in the myocardial infarction process.

### 3.2 miR‐128 targets MDFI to regulate the development of cardiac myocyte cell lines

**FIGURE 2:**
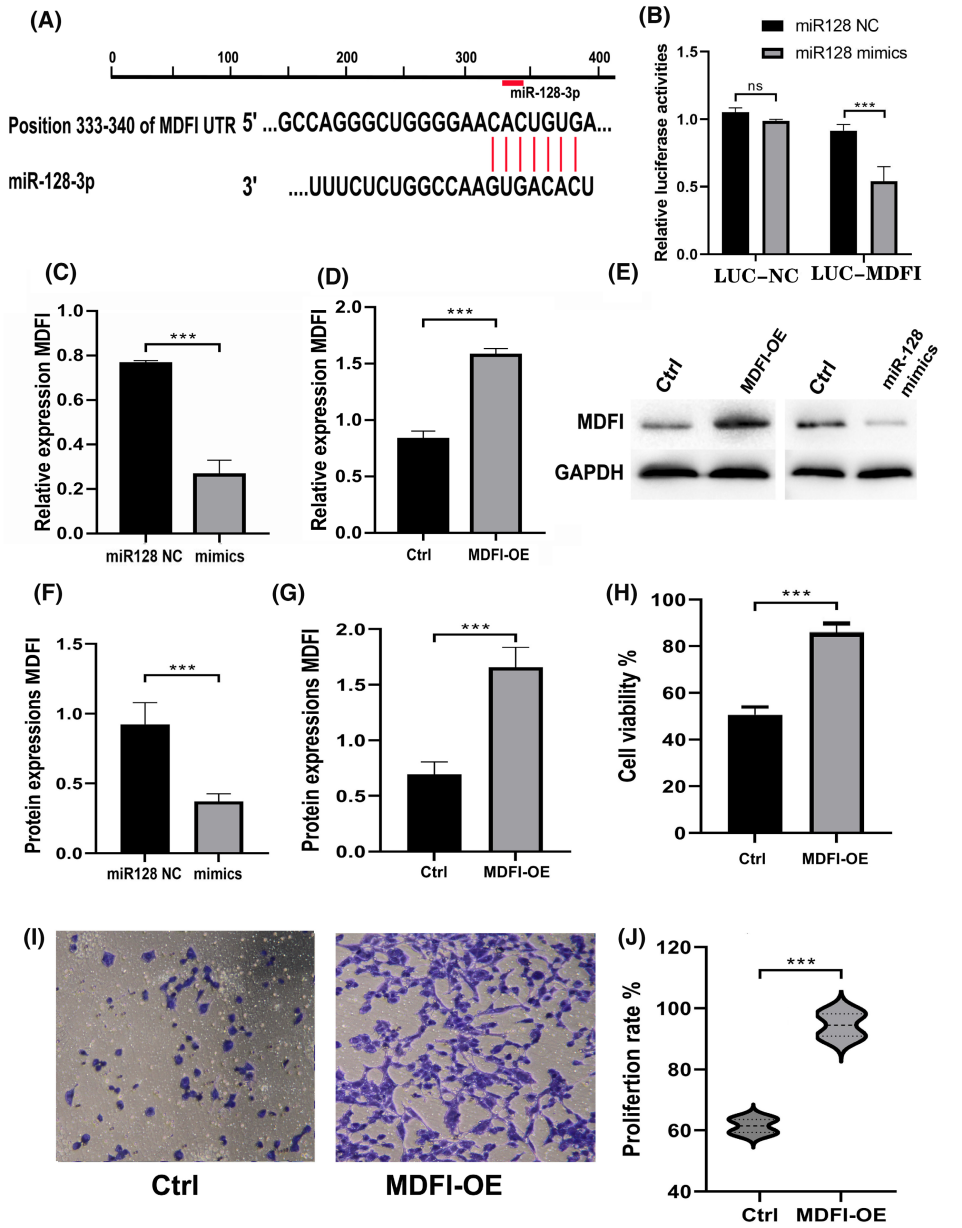
miR‐128 targets MDFI to regulate the development of cardiac myocyte cell lines. (A) The miR‐128‐3p and MDFI 3'UTR interaction sites, as forecasted by TargetScan; (B) dual luciferase reporter assay revealed reduced activated response in miR‐128‐3p mimics group on the MDFI gene; (C, D) qPCR showing upgraded expression of MDFI in cardiomyocytes treated with MDFI‐OE and the downregulated expression of MDFI in miR‐128‐mimics cells, respectively; (E–G) western blot results showing upgraded expression of MDFI in cardiomyocytes treated with MDFI‐OE and the downregulated expression of MDFI in miR‐128‐mimics transfected cells, respectively; (H–J) overexpression of MDFI promoted cell proliferation in MDFI‐OE group. Results are shown as mean ± SEM based on three replicates, with *n* = 3. Statistically, ***p < 0.001 denotes significance relative to the control group.

MDFI is a transcription factor that plays an important role in cardiomyocyte development. MDFI has been shown to be involved in the formation of the mature cardiomyocytes, as well as to regulate the expression of specific genes that are essential for cardiomyocyte differentiation. To investigate the relationship between miR‐128 and MDFI, we performed a Target‐scan analysis and the results confirmed that miR‐128 was positively correlated with MDFI on its position 333–340 3'UTR (Figure 2A). To further explore the functional relationship between miR‐128 and MDFI, we performed a luciferase reporter assay. The results showed that the luciferase activity was significantly decreased when the MDFI 3'UTR was co‐transfected with the miR‐128 mimic compared with the control group (Figure 2B). These results indicated that miR‐128 directly targets and regulates the expression of MDFI. Therefore, we performed both qPCR and western blotting to evaluate the expression profiling of MDFI in HL1 cells treated with both MDFI‐OE and miR‐128 mimics, respectively. Our results demonstrated that the expression of miR‐128 has an inhibitory effect on MFDI expression (Figure 2C–G). Moreover, the CCK8 results revealed that HL1 cells treated with MDFI‐OE displayed an improved percentage of cell viability and upgraded their proliferation in comparison with the control (Figure 2H–J). Thus, MDFI expression plays a key regulatory role in cardiac cell development, and controlling MDFI expression may serve as a therapeutic target for the treatment of cardiac dysfunction.

### 3.3 miR‐128 modulates Wnt/β‐catenin pathway through MDFI direct target

**FIGURE 3:**
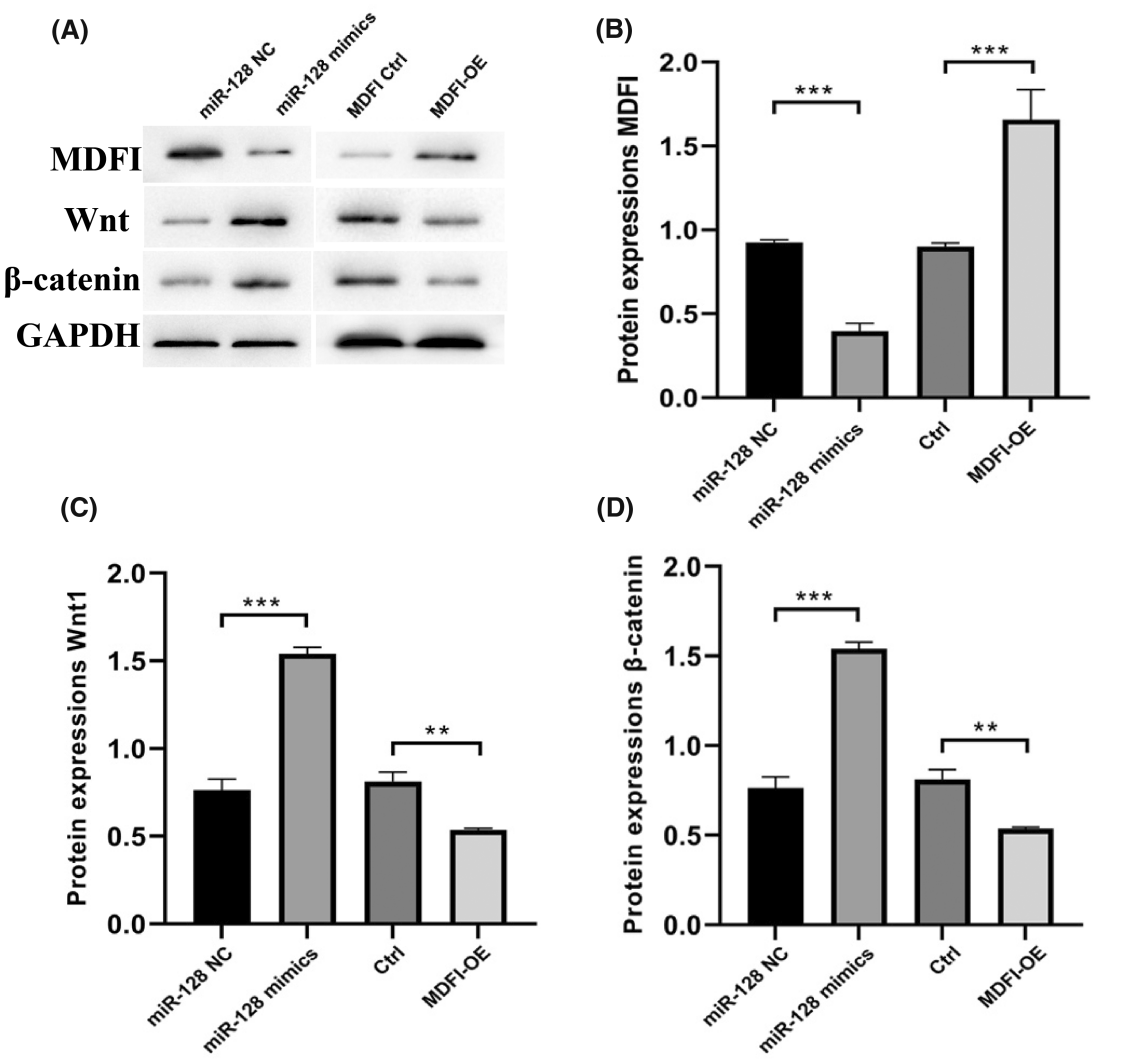
The interaction of miR‐128‐3p with the MDFI gene reveals a reciprocal relationship affecting the Wnt1/β‐catenin signalling in cardiomyocytes. (A) Protein expressions of MDFI, Wnt1 and β‐catenin visualized via western blot. (B) Relative protein quantities of MDFI, (C) Wnt1 and (D) β‐catenin were assessed by densitometry and normalized to GAPDH. Data represent mean SEM from three independent experiments, with *n* = 3 for each. Significance denoted by **p < 0.01, ***p < 0.001 versus control.

Wnt/β‐catenin pathway is thought to promote deleterious cardiac remodelling and functional decline. Therefore, HL1 cells were transfected with MDFI‐OE and miR‐128 mimics to evaluate the effects of MDFI expression on the Wnt/β‐catenin pathway; HL1 cells transfected with pCDH‐Empty vector and miR‐128 NC (negative control) were used as controls. The protein expressions of MDFI, Wnt1 and β‐catenin were assessed through western blot and the results indicated that the miR‐128 significantly suppressed the expression of MDFI (Figure 3A,B). Meanwhile, the overexpression of MDFI considerably suppressed the expressions of both Wnt1 and β‐catenin, while the cell treated with miR‐128 mimics displayed upgraded expressions of Wnt1 and β‐catenin. This opposite reaction confirmed that mirR‐128 negatively regulates the function of MDFI in cardiomyocytes (Figure 3C,D). Taken together, these data convincingly showed that MDFI could regulate the development of cardiomyocytes, and might have an essential role in the progression of HF by controlling the Wnt1/β‐catenin pathway through the down‐regulatory target of miR‐128.

### 3.4 Overexpression of MDFI disactivated the Wnt/β‐catenin signalling pathway

**FIGURE 4:**
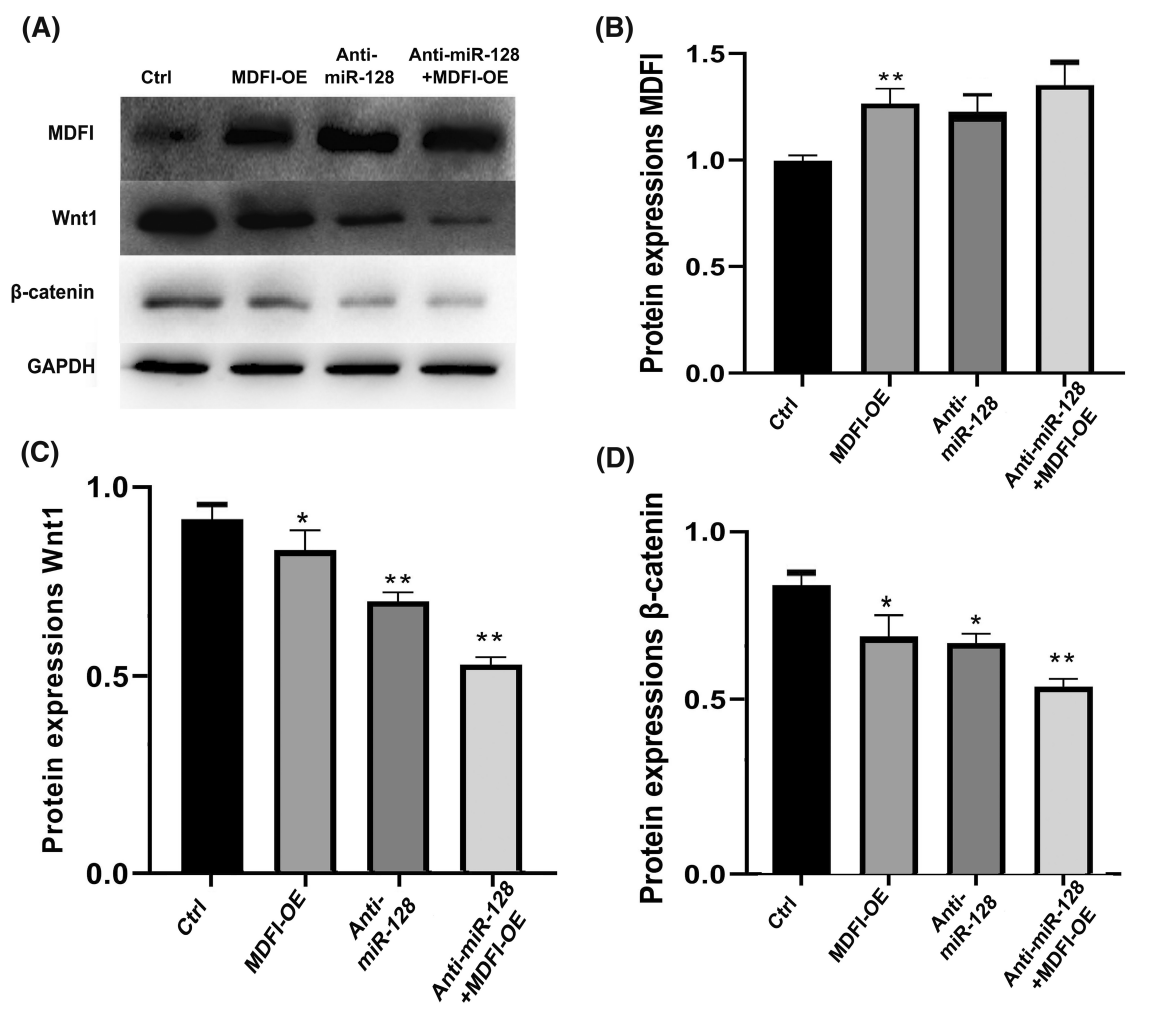
Inhibition miR128‐3p disactivated the MDFI/Wnt/β‐catenin signalling pathway. (A) The western blot panels show MDFI, Wnt1 and β‐catenin protein levels. (B) Relative expressions of MDFI, (C) Wnt1 and (D) β‐catenin were determined by densitometry, normalized against GAPDH. Data represent the mean ± SEM from three experiments, *n* = 3 per group. *p < 0.05, **p < 0.01 versus control.

Given miR‐128's pivotal role in modulating the Wnt1/β‐catenin pathway, we probed the regulatory effects of miR‐128 on MDFI within this signalling cascade in cardiomyocytes. To evaluate the influence of MDFI in cardiomyocytes, we over‐expressed MDFI (MDFI‐OE) and anti‐miR‐128. The substantial upsurge in MDFI levels upon miR‐128 inhibition validated its critical role in restraining MDFI expression. Conversely, concurrent miR‐128 inhibition and MDFI overexpression resulted in the downregulation of both Wnt1 and β‐catenin. Moreover, the simultaneous treatment with anti‐miR‐128 and MDFI‐OE demonstrated a cumulative decrease in Wnt1/β‐catenin levels, underscoring the antagonistic actions of miR‐128 and MDFI in the regulation of this pathway (refer to Figure 4A–D). These insights suggest that miR‐128 inhibition might bolster cardiomyocyte proliferation and heart regeneration by impeding the MDFI/Wnt1/β‐catenin axis, thus shielding cardiomyocytes from apoptosis and ensuring their regulation.

### 3.5 Loss of miR‐128 and overexpressing MDFI restored heart function in mice treated with ISO infusion

**FIGURE 5:**
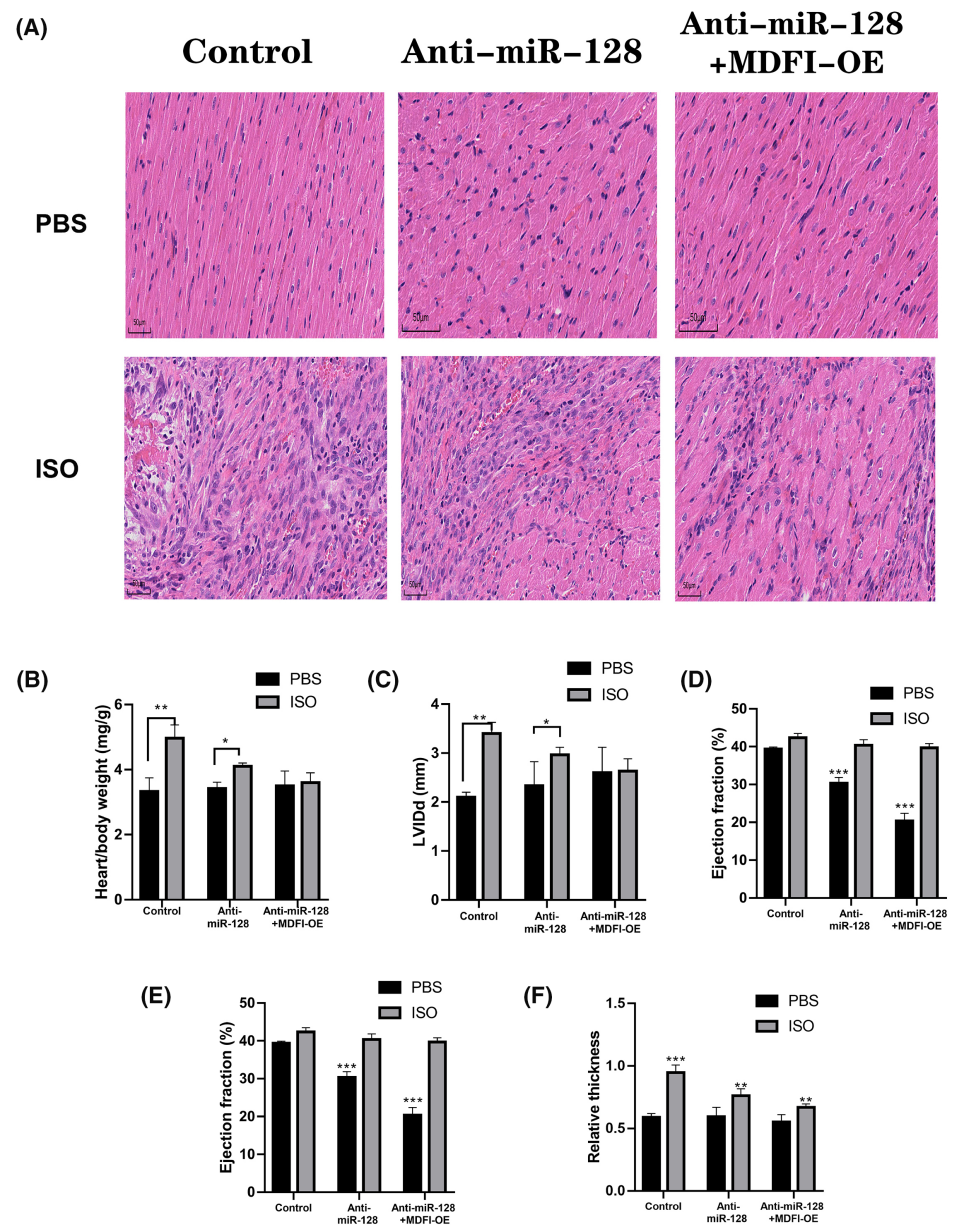
Restoration of heart function in mice treated with ISO infusion by anti‐miR‐128 and overexpression of MDFI. (A) Representative haematoxylin and eosin‐stained heart slices from mice. (B) Echocardiography results depicting heart/body weight ratio. (C) Left ventricular internal diameter (LVID), (D) fractional shortening, (E) ejection fraction and (F) wall thickness measurements. Data are presented as mean ± SEM with *n* = 3 for each group. **p < 0.01, ***p < 0.001 compared to the saline‐treated control group.

Subsequently, we delved into the pathological consequences of miR‐128 and MDFI interaction on cardiac hypertrophy and HF. We assessed hypertrophic changes and cardiac performance in both control and ISO‐stimulated mice, which received either anti‐miR‐128, a combination of anti‐miR‐128 and MDFI‐OE or no treatment at all. Histological analysis via haematoxylin and eosin staining revealed diminished hypertrophic manifestations in heart tissue cross‐sections of mice treated with anti‐miR‐128. Notably, heart sections from groups treated with anti‐miR‐128 alone or in conjunction with MDFI‐OE showed a preserved cardiomyocyte architecture and reduced hypertrophic indicators, particularly in ISO‐challenged hearts, compared to their untreated counterparts. Moreover, the augmentation of miR‐128 and concurrent suppression of MDFI did not adversely affect baseline cardiac function (Figure 5A). Comprehensive echocardiographic evaluation, including parameters such as heart‐to‐body weight ratio, left ventricular internal diameter (LVID), fractional shortening, ejection fraction and myocardial wall thickness, collectively suggested that MDFI‐OE mitigates hypertrophic alterations, leading to less extensive cardiac damage and improved cardiac performance (Figure 5B–F **)**. These findings advocate for the potential of miR‐128 and MDFI as a prophylactic approach against HF and the progression of myocardial hypertrophy.

## 4 DISCUSSION

MiR128‐3p plays a crucial role in regulating the growth and maintenance of cardiomyocytes. Studies have shown that miR128‐3p is involved in the regulation of the proliferation and differentiation of cardiomyocytes and the maintenance of cardiomyocyte function.^15^, ^16^ A recent study suggested that miR128 targets important cell cycle‐related genes to prevent the growth of postnatal cardiomyocytes. Increased expression of miR128‐3p in the myocardium in response to ischemia shock was at least partially responsible for insulin resistance.^17^ Despite being under hypoxic stress, deletion of miR128‐3p allowed insulin‐dependent glucose absorption to continue. As a result, miR128‐3p was suggested to be actively involved in myocardial glucose metabolism. Additionally, as glucose uptake promotes cardiomyocyte proliferation in the early stages of development, this finding adds to the rationale for earlier findings that miR128‐3p suppression promotes postnatal and adult cardiac regeneration.^16^, ^18^ Herein, the overexpression of miR‐128 promoted the apoptosis of cardiomyocytes and impeded their proliferation, confirming that miR128‐3p is a key regulator actor of cardiomyocyte development.

The Wnt/β‐catenin pathway is important for regulating cell proliferation, differentiation and gene expression and is essential for cardiac development and maintenance.^19^, ^20^ In HF, the activation of the Wnt/β‐catenin pathway is thought to promote deleterious cardiac remodelling and functional decline. Studies have shown that increased Wnt/β‐catenin activity is associated with increased fibrosis, hypertrophy and apoptosis, causing decreased contractility and cardiac output.^21^ In addition, the Wnt/β‐catenin pathway is involved in the pathogenesis of ventricular arrhythmias and is thought to be a major contributor to the progression of HF.^22^ Numerous studies have proposed various strategies to target the Wnt/β‐catenin pathway in HF.

MDFI, a compact molecule, has been found to obstruct the MyoD family of transcription factors' activity, a group implicated in cardiac muscle development regulation.^23^ By inhibiting the activity of these factors, MDFI has been shown to reduce the rate of HF progression by decreasing the amount of fibrosis and inflammation, as well as improving cardiac contractility. Additionally, MDFI has been shown to reduce the expression of pro‐fibrotic genes and to increase the expression of anti‐fibrotic genes.^24^ Controversially, in tumour cells, the inhibition of MDFI attenuates the proliferation and glycolysis of Helicobacter pylori‐infected gastric cancer cells by inhibiting Wnt/β‐catenin pathway.^25^ While in our experiment, MDFI inhibition resulted in decreased Wnt/β‐catenin protein level.

As the effect of miR128‐3p overexpression on MFDI expression is not known yet. In this study we demonstrated that miR‐128 mimics significantly inhibited the expression of MDFI in cardiomyocyte. Also, the overexpression of MDFI considerably decrease the cardiomyocyte proliferation by inhibiting the Wnt/β‐catenin pathway. Therefore, targeting miR218/MDFI may provide a novel therapeutic approach for treating HF. While, larger sample size and more systemic work need to be done to improve and validate the functionality of miR218, in order to ultimately promote its clinical application.

## 5 CONCLUSION

To conclude, our research offers substantial proof of miR‐128 and MDFI's combined molecular interaction, playing a significant role in the onset of cardiac hypertrophy and HF. Our findings reveal a previously unidentified, twofold stimulatory role of miR‐128 that promotes the Wnt/β‐catenin pathway activation, closely linked with the progression of cardiac hypertrophy and eventual HF. The insights garnered from our investigation suggest that the suppression of miR‐128, coupled with the augmented expression of MDFI, may herald new, effective therapeutic strategies, broadening the spectrum of interventions for combating HF.

## AUTHOR CONTRIBUTIONS

**Sun Yanjun:** Data curation (lead); formal analysis (equal); investigation (lead); methodology (equal); visualization (equal); writing – original draft (lead); writing – review and editing (equal). **Gu Yunfen:** Methodology (equal); project administration (equal); resources (equal); validation (equal); writing – review and editing (equal). **Yao Haoyi:** Methodology (equal); validation (equal). **Wang Zhe:** Methodology (equal); resources (equal). **Qiu Jiapei:** Conceptualization (lead); funding acquisition (lead); resources (equal); validation (equal); writing – review and editing (equal).

## CONFLICT OF INTEREST STATEMENT

The authors declare no conflict of interest.

## Data Availability

The data that support the findings of this study are available from the corresponding author upon reasonable request.
